# Laparoscopic-assisted distal gastrectomy and central pancreatectomy for gastric and perigastric lymph node metastases and pancreatic invasion from melanoma: a case report

**DOI:** 10.1186/s40792-020-01027-0

**Published:** 2020-09-29

**Authors:** Yuki Okawa, Yuma Ebihara, Kimitaka Tanaka, Yoshitsugu Nakanishi, Toshimichi Asano, Takehiro Noji, Yo Kurashima, Soichi Murakami, Toru Nakamura, Takahiro Tsuchikawa, Keisuke Okamura, Toshiaki Shichinohe, Satoshi Hirano

**Affiliations:** Department of Gastroenterological Surgery II, Faculty of Medicine, Hokkaido University, Kita 15 Nishi 7, Kita‐ku, Sapporo, 060‐8638 Japan

**Keywords:** Malignant melanoma, Central pancreatectomy, Laparoscopic distal gastrectomy

## Abstract

**Background:**

In melanoma, completely resectable metastases are surgically resected to expect to prolong relapse-free survival and overall survival. However, distant metastases of melanoma are rarely indicated for surgery because multiple metastases are often observed at diagnosis. We report a case of a man in his 50s who underwent laparoscopic-assisted distal gastrectomy and central pancreatectomy for gastric metastases, lymph node metastases, and pancreatic invasion that could be completely resected.

**Case presentation:**

A 50-year-old man was diagnosed with malignant melanoma of the left parietal region. After diagnosis, tumor resection and left cervical lymph node dissection were performed, and interferon-β treatment was added as adjuvant therapy. Seventeen months after adjuvant therapy, metastasis of stomach and abdominal lymph nodes from melanoma was diagnosed. And the pancreatic invasion of lymph nodes was suspected. Laparoscopic-assisted distal gastrectomy and the central pancreatectomy were performed because pancreatic invasion of melanoma was intraoperatively found. After 9 months of relapse-free survival, abdominal recurrence was observed. Nivolumab and ipilimumab were administered, and recurrent lesions are currently controlled. The patient has survived more than 3 years since metastasis resection.

**Conclusion:**

In conclusion, laparoscopic-assisted distal gastrectomy and the central pancreatectomy were performed for gastric and perigastric lymph node metastases and pancreatic invasion due to malignant melanoma, and the negative surgical margin was achieved. Although patient selection is required, the central pancreatectomy was a good indication for maintaining exocrine and endocrine function. The development of immune checkpoint inhibitors and molecular-targeted agents may increase gastrointestinal surgery for metastatic melanoma in the future.

## Background

Melanoma with distant metastases is a malignant neoplasm with a poor prognosis. In melanoma, completely resectable metastases, so-called oligometastases, are surgically resected to expect to prolong relapse-free survival and overall survival [1]. However, in most cases with metastatic melanoma, multiple metastases are present at the time of diagnosis and are rarely indicated for surgery.

Gastric metastasis of malignant melanoma is asymptomatic and its diagnosis rate is low. The prevalence of gastric metastasis of malignant melanoma diagnosed at autopsy is 20–40%, but it is rare to be diagnosed before death, despite improved imaging and endoscopy diagnostic capabilities [2, 3]. In addition, because melanoma metastasis to the pancreas accounts for only 2.3% of all abdominal organs [4], simultaneous gastrectomy and pancreatectomy for metastatic melanoma is rare. We report a case of a man in his 50 s who underwent laparoscopic-assisted distal gastrectomy and central pancreatectomy for gastric metastases, lymph node metastases, and pancreatic invasion that could be completely resected.

## Case presentation

**Fig. 1:**
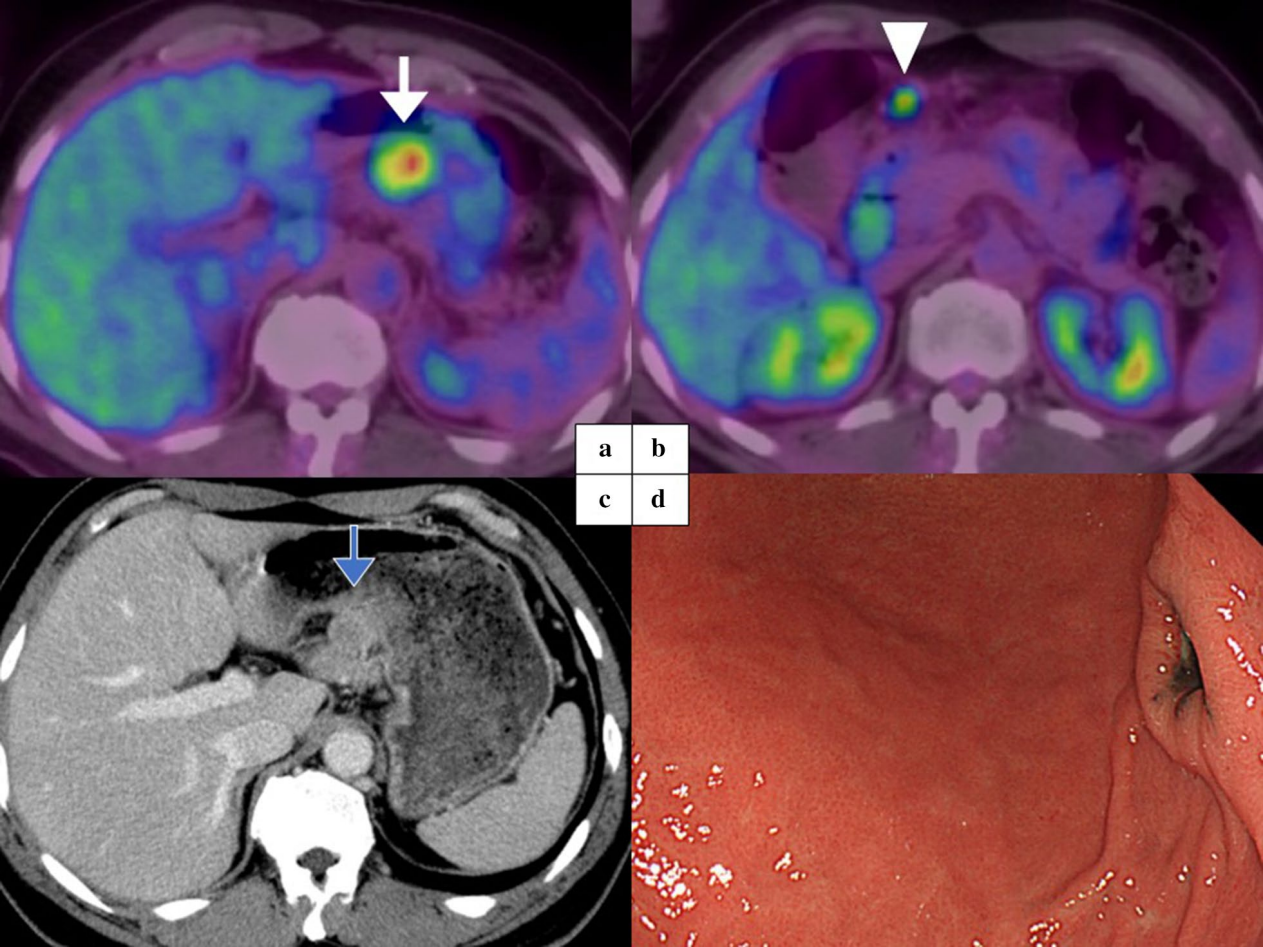
Images of preoperative examinations. **a**, **b** The PET–CT images. The white arrow shows uptake around the lymph nodes along the lesser curvature. The white arrowhead shows uptake around subpyloric lymph nodes. **c** The e-CT image. The blue arrow shows the lymph nodes along the lesser curvature may have invaded the pancreas. **d** The EGD revealed a 20 mm submucosal tumor-like lesion with a melanotic ulcer on the posterior wall of the gastric angle. PET–CT: fluorodeoxyglucose positron emission tomography and computed tomography, e-CT: enhanced computed tomography, EGD: esophagogastroduodenoscopy

A 50-year-old Japanese man was diagnosed with malignant melanoma of the left parietal region. Tumor resection and left cervical lymph node dissection were performed. The pathological diagnosis was pT4aN1aM0 pStage IIIC with Breslow's thickness of 8.6 mm and no ulcer. Interferon-β therapy was added as adjuvant therapy. Seventeen months after adjuvant therapy, the fluorodeoxyglucose positron emission tomography and computed tomography (PET–CT) showed uptake around the lymph nodes along the lesser curvature and subpyloric lymph nodes (Fig. 1a, b). The enhanced computed tomography (e-CT) revealed that the lymph nodes along the lesser curvature may have invaded the pancreas (Fig. 1c). Esophagogastroduodenoscopy (EGD) revealed a 20 mm submucosal tumor-like lesion with a melanotic ulcer on the posterior wall of the gastric angle (Fig. 1d). A biopsy of the submucosal tumor identified the lesion as melanoma.

Based on the above examination, metastasis of stomach and abdominal lymph nodes from melanoma was diagnosed. And the pancreatic invasion of lymph nodes was suspected. No distant metastases to the other were noted. It was determined that surgery could completely remove the metastatic lesions. Distal gastrectomy was planned, and pancreatectomy was planned to add as needed.

**Fig. 2:**
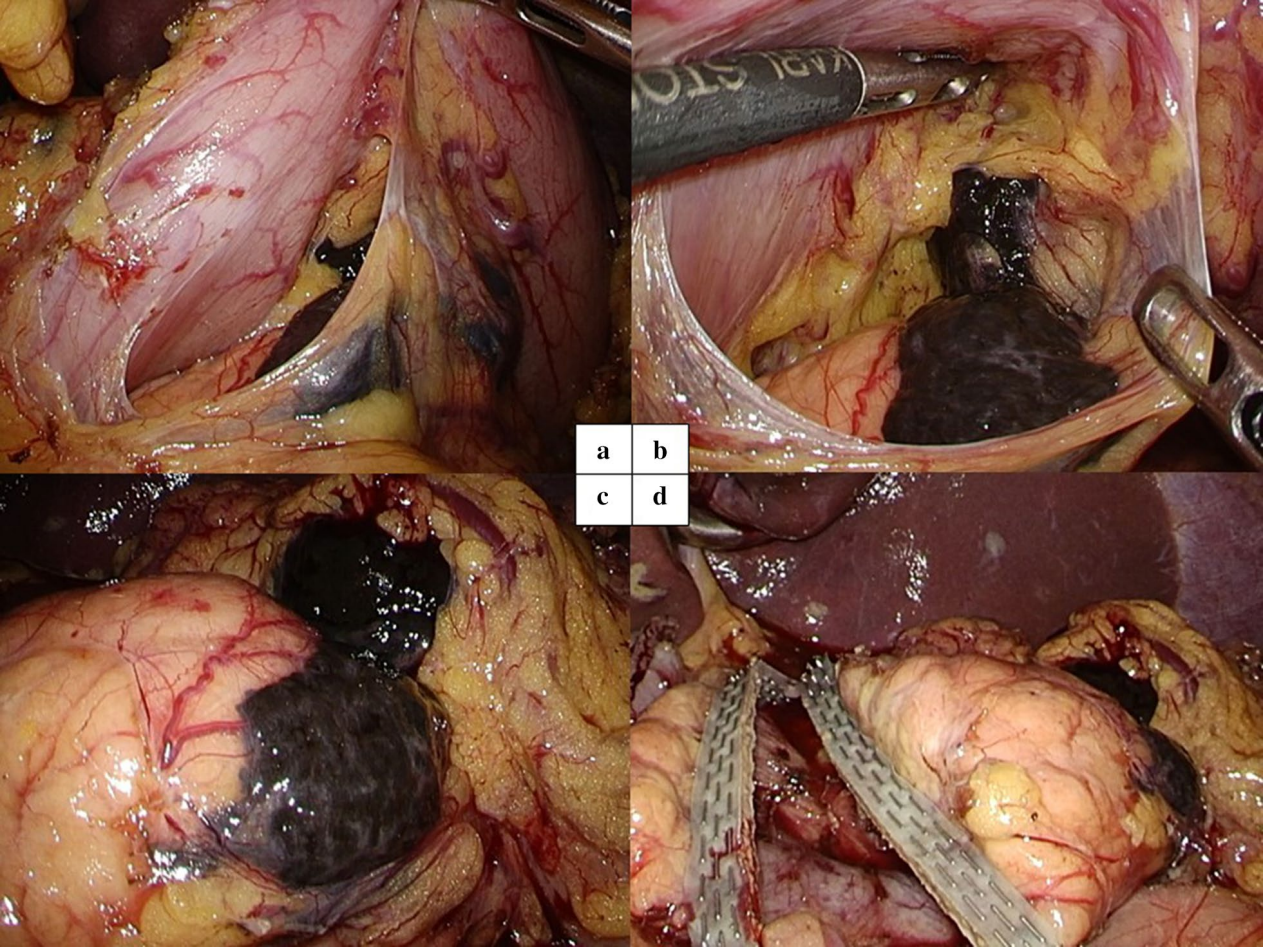
Intraoperative images of laparoscopic surgery. **a** Lifting the stomach confirmed black lymph nodes along the lesser curvature. **b** The metastatic area of melanoma spread to the pancreas. **c** After peeling around the pancreas, a clear boundary between the tumor and normal pancreas was confirmed. **d** The pancreas was divided above the portal vein using a linear stapler. The margin with the tumor boundary was secured

First, laparoscopic surgery was performed. No peritoneal metastasis was noted. In opening the omental bursa, black lymph nodes along the lesser curvature were found (Fig. 2a, b). Black lymph nodes invaded the body of the pancreas, but the boundaries were clear (Fig. 2c). No other metastases were confirmed, and it was considered that complete resection was possible. Laparoscopic distal gastrectomy was performed with lymph node dissection.

**Fig. 3:**
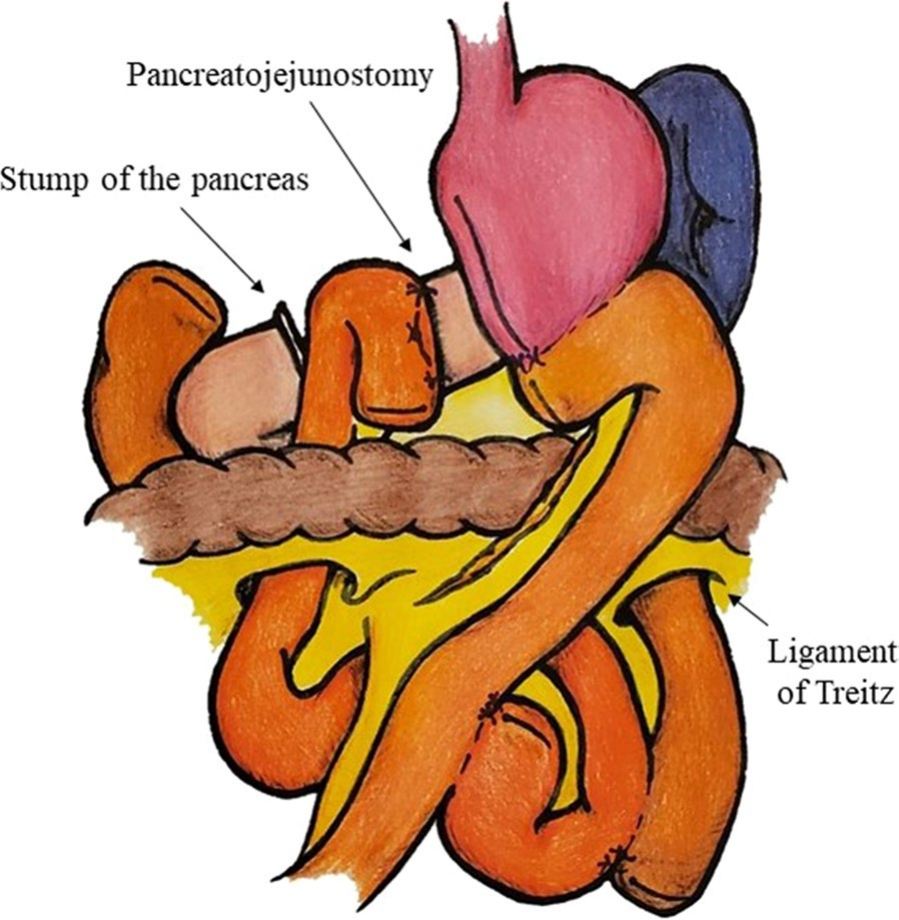
The illustration of the reconstruction. Roux-en-Y reconstruction was performed, and four anastomoses were performed: gastrojejunostomy, pancreaticojejunostomy and two jejunojejunostomies

Second, the dorsal side of the pancreas was separated from the superior mesenteric vein and the pancreas was divided above the portal vein using a linear stapler (Fig. 2d). The metastasis resection was completed by laparoscopic surgery, but the procedure was shifted to open surgery for gastrointestinal reconstruction. A midline incision was made in the epigastric wall. The distal pancreas was dissected, and a specimen was extracted. Roux-en-Y reconstruction was performed, and four anastomoses were performed: gastrojejunostomy, pancreaticojejunostomy and two jejunojejunostomies (Fig. 3). The operation time was 543 min and blood loss were 200 ml.

**Fig. 4:**
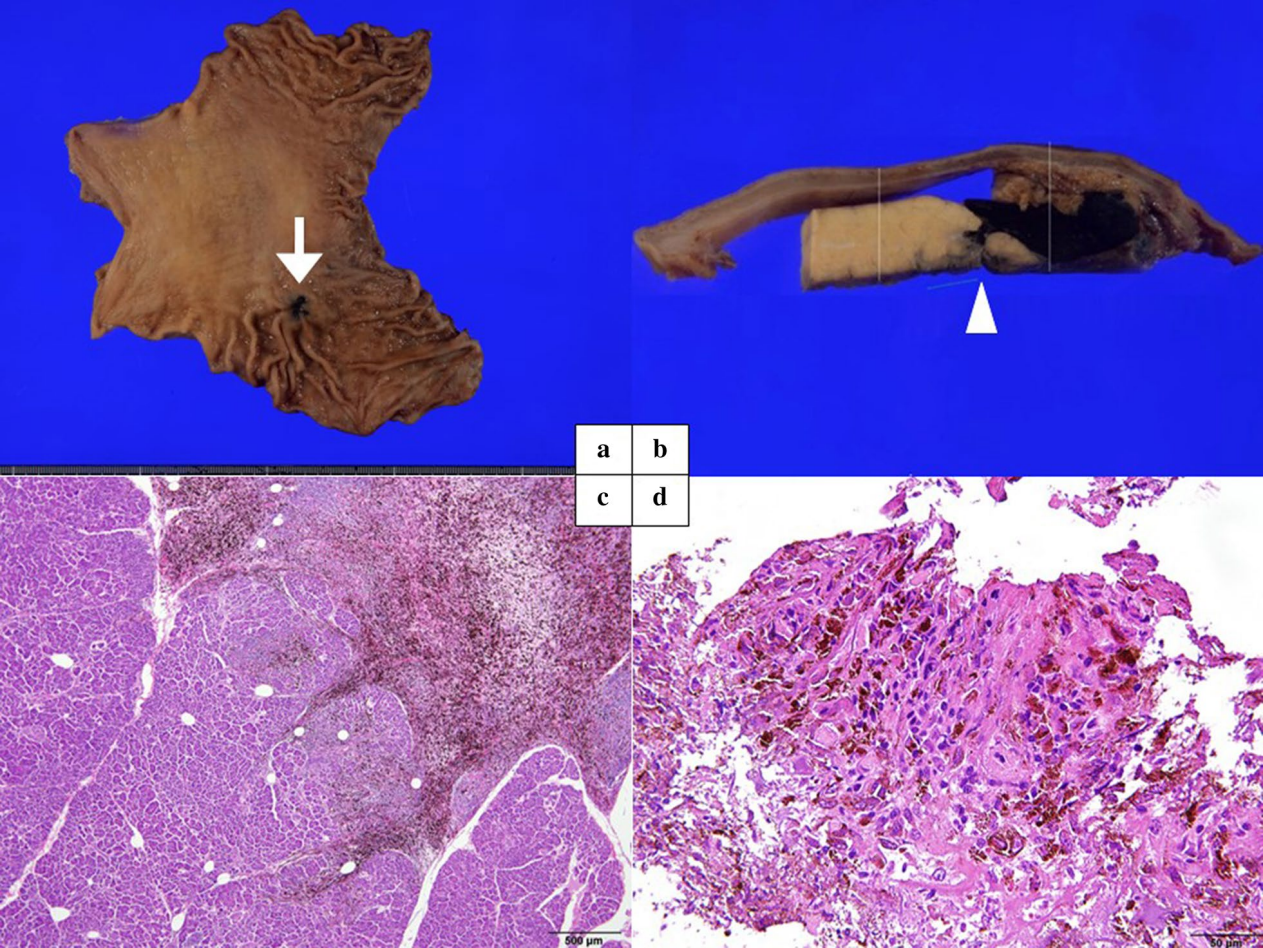
Macro and microscopic findings of the resected specimen. **a** Macroscopic findings of a stomach specimen. The white arrow shows submucosal tumors with melanocytic ulcers. **b** Macroscopic findings of the pancreatic body specimen divided into sagittal sections. The white arrowheads show smooth melanin-like solid lymph nodes with distinct borders invading the pancreas. **c**, **d** Microscopic findings of invaded pancreas. There was solid growth of tumor cells with distinct eosinophilic vesicles and nucleoli, many of which contained melanin granules

The specimens showed submucosal tumors with melanocytic ulcers (Fig. 4a), as well as smooth melanin-like solid lymph nodes with clear demarcation lines that invade the pancreatic body (Fig. 4b). Microscopic examination showed solid growth of tumor cells with distinct eosinophilic vesicles and nucleoli, many of which contained melanin granules (Fig. 4c, d). Immunohistochemical analysis revealed that the lesions were positive for the melanoma-specific antigens Melan-A and HMB-45 and negative for S-100. The stomach and pancreas margins were negative.

Postoperatively, delayed gastric emptying was observed, but no other complications of Clavien–Dindo III or higher were observed. Abdominal recurrence was observed after 9 months of relapse-free survival. Nivolumab and ipilimumab were administered, and recurrent lesions are currently controlled. The patient has survived more than 3 years since metastasis resection.

## Discussion

Melanoma is a malignant neoplasm that causes hematogenous or lymphatic metastatic growth. When distant metastatic lesions of melanoma are found, surgery is rarely indicated because multiple metastases are often observed. Whereas, a few metastases that can be completely excised, so-called oligometastases, have a long relapse-free survival and over survival after resection [5, 6]. Excision of the gastrointestinal tract and pancreas has been reported to prolong overall survival compared to non-excision [4].

The diagnosis rate of gastric metastasis of melanoma is low because it is asymptomatic, although the prevalence of gastric metastasis of malignant melanoma diagnosed at autopsy is 20–40% [2, 3]. Furthermore, in a report summarizing cases of melanoma abdominal organ metastases, only 2.3% of all abdominal organs had pancreatic metastases [4]. From these reports, simultaneous resection of metastatic melanoma lesions spanning the stomach and pancreas is a rare case.

There is no provision for lymph node dissection in metastatic melanoma in the gastrointestinal tract. The patient underwent a distal gastrectomy with the same extent of lymph node dissection for gastric cancer with sufficient margin to the tumor to prevent dissemination. The feasibility of local resection and metastatic lymph node dissection should be evaluated by accumulating future cases.

A pancreatectomy was required to completely remove the lymph nodes along the lesser curvature that invaded the pancreas. Since the invasive site in this case was the body of the pancreas, spleen-preserving distal pancreatectomy and central pancreatic resection were given as options. Both procedures can treat pancreatic lesions to maintain the spleen and ensure adequate blood flow to the residual stomach, but the residual pancreatic volume is greater in central pancreatectomy. A meta-analysis comparing central pancreatectomy with spleen-preserving distal pancreatectomy revealed that central pancreatectomy had a lower risk of endocrine and exocrine dysfunction [7]; whereas, it was also revealed that pancreatic fistula often occurs in the central pancreatectomy, and the selection of the surgical method for each patient is important. Since the patient in this case was young in his 50s, it was considered necessary to maintain both endocrine and exocrine pancreatic function.

With the recent development of immune checkpoint inhibitors and molecular-targeted agents, the prognosis can be expected to improve even for malignant melanoma with metastases by combining these novel drug therapies with surgery. In fact, the usefulness of nivolumab as postoperative adjuvant therapy after metastatic excision has been verified [8]. In this case, the relapse-free survival was 9 months, but the patient has survived for more than 3 years on a combination of molecularly targeted drugs and immune checkpoint inhibitors after metastasis resection. Although there are few metastatic melanomas that are indicated for surgery due to the large number of multiple metastases at the time of diagnosis, the number of surgical cases such as this case may increase in combination with novel drug therapy.

## Conclusion

In conclusion, laparoscopic-assisted distal gastrectomy and the central pancreatectomy were performed for gastric and perigastric lymph node metastases and pancreatic invasion due to malignant melanoma, and the negative surgical margin was achieved. Although patient selection is required, the central pancreatectomy was a good indication for maintaining exocrine and endocrine function. The development of immune checkpoint inhibitors and molecular-targeted agents may increase gastrointestinal surgery for metastatic melanoma in the future.

## Data Availability

The datasets supporting the conclusions of this article are included within the article.

## Abbreviations

PET–CT: Fluorodeoxyglucose positron emission tomography and computed tomography
e-CT: Enhanced computed tomography
EGD: Esophagogastroduodenoscopy
